# Tissue-Mimicking Geometrical Constraints Stimulate Tissue-Like Constitution and Activity of Mouse Neonatal and Human-Induced Pluripotent Stem Cell-Derived Cardiac Myocytes

**DOI:** 10.3390/jfb7010001

**Published:** 2016-01-07

**Authors:** Götz Pilarczyk, Alexandra Raulf, Manuel Gunkel, Bernd K. Fleischmann, Robert Lemor, Michael Hausmann

**Affiliations:** 1 Kirchhoff Institute für Physik, Im Neuenheimer Feld INF 270, Heidelberg D-69120, Germany; hausmann@kip.uni-heidelberg.de; 2 Institut für Physiologie der Unversität Bonn, Life & Brain Center, Sigmund Freud Strasse 25, Bonn D-53127, Germany; araulf@uni-bonn.de (A.R.); Bernd.Fleischmann@uni-bonn.de (B.K.F.); 3 ViroQuant Cell Networks RNAi Screening Facility, BioQuant Center, Im Neuenheimer Feld INF 267, Heidelberg D-69120, Germany; manuel.gunkel@bioquant.uni-heidelberg.de; 4 Luxembourg Institute for Science and Technology, 5 avenue des Hauts-Fourneaux, Esch-Belval L-4362, Luxembourg; robert.lemor@list.lu

**Keywords:** hIPSC-derived cardiac myocytes, surface pattern, cardiac tissue engineering, stem cells, structure-based cardiac arrhythmia

## Abstract

The present work addresses the question of to what extent a geometrical support acts as a physiological determining template in the setup of artificial cardiac tissue. Surface patterns with alternating concave to convex transitions of cell size dimensions were used to organize and orientate human-induced pluripotent stem cell (hIPSC)-derived cardiac myocytes and mouse neonatal cardiac myocytes. The shape of the cells, as well as the organization of the contractile apparatus recapitulates the anisotropic line pattern geometry being derived from tissue geometry motives. The intracellular organization of the contractile apparatus and the cell coupling via gap junctions of cell assemblies growing in a random or organized pattern were examined. Cell spatial and temporal coordinated excitation and contraction has been compared on plain and patterned substrates. While the *α*-actinin cytoskeletal organization is comparable to terminally-developed native ventricular tissue, connexin-43 expression does not recapitulate gap junction distribution of heart muscle tissue. However, coordinated contractions could be observed. The results of tissue-like cell ensemble organization open new insights into geometry-dependent cell organization, the cultivation of artificial heart tissue from stem cells and the anisotropy-dependent activity of therapeutic compounds.

## 1. Introduction

Current advances in stem cell research provide a cell repertoire that facilitates the artificial reconstitution of multicellular ensembles with properties close to native tissues [1]. While lineage selection, directed and functional differentiation of pluripotent stem cells are progressively elucidated on a broad basis, the tissue re-organization process is still relatively little developed. This may be due to the general problems of controlling cell division and growth in tissue-shaped environments and of inducing appropriate cell to cell interactions, including tissue-like cell connections, in order to establish native intercellular signaling. Whereas any cell type, regardless of whether being primarily cultured after collection from vendor tissue or expanded in permanent culture lines, is randomly shaped and contacted due to the culture dish environment, cell to cell contacts are consequently random or affected by locally-restricted stimuli. Such approaches are useful for tissue engineering in the case that the disorganization of such tissue reconstructs is acceptable, for example artificially-grown tumors in radiation research or pharmaceutical testing.

Alternatively, the application of a stable chemical or physical stimulus gradient is suitable to induce a graded cell differentiation, as in the case of skin models. In that case, an organized cell ensemble is formed by cell expansion in a differentially-humidified volume superposed by a local cell division zone on one end with one-directional cell differentiation towards an opposed end representing the skin surface periphery [2,3]. A skin reconstitution organized by such gradients lacks higher-order cell ensemble organization, like paracrine-controlled pigment distribution, thickness regulation by cytokine-controlled cell proliferation and local activity of cell types, like fibroblasts [4,5].

A further step towards the generation of tissue-resembling complexity is the culture of cells in scaffolds, providing cell growth, adhesion and migration under supportive physical and geometrical constraints [6]. Such guiding structures can consist of porous bulk material, or extracellular matrix (ECM) depositions, of extruded or printed cell carriers, or cell-loaded matrices [7,8,9,10,11]. The sole restriction of environmental space is sufficient in some cases to stimulate differentiation programs and to provoke the expression of proteins characteristic for a particular cell type [12,13,14].

The engineering of tissue with high spatial regularity, but a reduced set of cell types, like skeletal or cardiac muscle tissue, can be performed on regularly-patterned surfaces or micro-structured scaffold bulks [15]. Such reconstitutions imitate structural, as well as functional key properties of the represented tissue [16]. The sheets are stacked by a method called cell sheet engineering to make up three-dimensional tissue patches for implantation [17].

The use of free spanning polydimethylsiloxane (PDMS) scaffolds was reported recently to introduce aspects of mechanical flexibility into the settlement and entrainment of well-organized macroscopic cardiac tissue sheets [18]. In this context, differentiation from stem cells and the resulting physiological properties are of concern [19,20]. The inclusion of cardiac myocytes into scaffolds by the use of combined stereolithography printing and micropatterning also addresses issues in micro-device manufacturing distinct from topics of cardio-regeneration [21,22].

Proteome examination of C2C12 mouse skeletal muscle cells grown under organizing cues *versus* cells grown in non-organizing environments reveals a remarkable number of proteins differentially expressed in dependency of the environmental organization state [23]. In the study presented here, we compared the development and differentiation of stem cells on non-patterned surfaces and in the presence of geometrical constraints; the latter suggested a tissue-like cell development. Surface patterning was performed in poly-dimethylsiloxane (PDMS), a two-component heat casting rubber gum with properties making it ideal for microscopy and cell culture. PDMS is colorless and highly transparent. It owns the same refraction index as microscopy glass carriers. It is of high chemical resistivity, but can be plasma etched to introduce hydroxyl groups for chemical coupling [24].

In this manuscript, we reconstitute a well-organized ensemble of differentiated cardiac myocytes to setup a thin tissue slice with the organization properties, the excitation spread and the coordinated contraction closely to terminally-differentiated ventricular tissue. We use two types of cardiomyocyte cell types. The first is derived from newborn mouse heart ventricles, thus representing heart cells differentiated *in vivo*. This conventional cell source is compared to hIPSC-derived cardiac myocytes, which are currently in use as stem cell-derived substitutes for transient cardiac myocyte cultures and heart tissue slices in, e.g., pharmaceutical compound testing [25].

The working hypothesis concerns the correlation of the spatial organization and physiological function of multicellular ensembles and native tissues: is it possible to constitute a tissue-like ensemble with extracellular orientation, intracellular organization and ensemble function close to the corresponding native tissue by cultivating vital multicellular populations under geometric constraints? Since several, but not all, signaling pathways and functions are expected to differ in cells upon cultivation on patterned substrates from those cultivated on plain substrates, we select four appropriate measurement indicators, each in reference to the geometric properties of the artificial environment. The indicators are visualized by confocal microscopy (confocal laser scanning microscopy (CLSM)) and scanning electron microscopy (SEM). The cardiac-specific contractile apparatus is stained by *α*-actinin and the intercellular electrical signal spread across gap junctions by connexin-43. These are key components of cell shape, cell contraction and signal conduction. In addition, a nuclear stain facilitates the estimation of the cell density. We assess the multi-cellular ensemble organization, the individual cell orientation, the cell interaction and communication and the intracellular organization of the contraction apparatus and of electrical signal transduction. A visualization of the concerted contraction dynamics of the cell ensemble as a result of the indicators listed above is also given.

For high cell density in combination with a high anisotropic cell orientation, the electrical coupling of cardiac myocytes inside regular patterned environments facilitates the optical examination of coordinated ensemble excitation activity. In this context, the signal spread across a small number of organized cells might uncover an immediate hand-over of electrical stimulation, not only via canonical super-threshold action potential generation, but also via non-canonical field fluctuations [26].

## 2. Experimental Section

### 2.1. PDMS Micro-Patterning on Glass Cover Slides: Geometry-Patterned Surfaces

If not otherwise stated, all chemicals are from Sigma-Aldrich Chemie GmbH, Munich, Germany. Sylgard 184 polydimethylsiloxane (PDMS) polymer and FluoroDish cell culture dishes were purchased at World Precision Instruments Germany GmbH, Berlin, Germany. Wafer mask development and wafer dry etching are remittance work according to the authors’ design sketches performed by the Fraunhofer IBMT, St. Ingbert, Germany. The design describes a micro-pattern of alternating 5 *μ*m-wide fillets and 30-*μ*m chamfers in a parallel 35-*μ*m periodicity with a length of 12 mm. After replication molding, the width ranges, depending on the gum polymer viscosity, from 5 to 10 *μ*m at the top of the fillets. The chamfers are of a crescent shape with a maximum diameter of 25–30 *μ*m at the upper face. The parallel fillet to chamfer pattern set up a field of 12 × 12 mm with an area of 144 mm${}^{2}$.

The two-step pattern casting procedure is derived by modifying a protocol published elsewhere [27]. For silicone replica molding, the wafers are cut into rectangular pieces of a 12-mm side length. After rinsing in acetone and ambient drying, the release coating is applied to the silicon surface: the acetone-rinsed wafer piece is cleaned in peroxymonosulfuric acid (*i.e.*, Caro’s acid), which is freshly prepared by mixing equal aliquots of concentrated sulfuric acid and hydrogen peroxide on ice. After 12 h in Caro’s acid, the silicon is rinsed in demineralized water and dried at 50 ${}^{\circ }$C. After reaching room temperature, the silicon is immediately transferred to silanization solution, Type 1 (4% dichlorodimethylsilane in n-heptan), for 1 h. After drying and cooling down to −20 ${}^{\circ }$C, the silicon surface is allowed to catch air moisture for hydrolyzing the chlorosilane. A subsequent period at 95 ${}^{\circ }$C for 12 h induces the condensation of the surface silanolic residues with moisture-derived dihydroxydimethylsilane, and a covalently-bound hydrophobic surface coating is deposited. The coated silicon matrix is mounted in a house-made brass holder with a molding cavity resulting in 1–2 mm-thick molds and a 13-mm square base area carrying the replicated micro-pattern. For improved polymer release, the holder surface is rubbed with household dish liquid. The Sylgard 184 polymer components are processed according to manufacturer recommendations and filled to the holder with the silicon matrix. After curing for a minimum 2 h at 95 ${}^{\circ }$C, the PDMS positive replica is peeled off manually. To get a negative PDMS replica, the PDMS-positive replica needs to be release coated. The positive replica is washed in ethanol p. a. (pro analysi or analytical grade) for a minimum of 72 h to remove low molecular weight siloxane compounds. The PDMS surface is converted into pure silica glass, as described elsewhere [28]. After oxygen plasma cleaning and surface activation of the polymer, the resulting glass surface is release coated in 96% ethanol p. a. with 5% heptadecafluoro-1.1.2.2.-tetrahydroxydecyl-trichlorosilane (HTTS, ABCR, Karlsruhe, Germany) for 24 h. A subsequent rinse in demineralized water converts the chloro- into hydroxyl-residues. The positive replicas are kept at 95 ${}^{\circ }$C for 12 h to condensate hydroxyl-residues to produce a super hydrophobic release coating. For negative replica molding, FluoroDishes are cleaned in Caro’s acid, as described above. A 100-*μ*L aliquot of curable PDMS is pressed between the negative mold and the dish cover slide bottom for 6 hours at 65 ${}^{\circ }$C, and the negative mold is peeled off by hand. For increased wettability, the dishes are washed in ethanol p. a. for 72 h and 15 nm of titanium coated in a plasma coater. A 10-*μ*g/mL fibronectin solution (No. F1141-1MG, Sigma-Aldrich Chemie GmbH, Munich, Germany) in demineralized water is used to coat the titanium surface for 12 h at 4 ${}^{\circ }$C.

### 2.2. Cell Culture

For isolation of primary neonatal mouse cardiac myocytes, the Neonatal Heart Dissociation Kit and the Neonatal Cardiomyocyte Isolation Kit for mice (No. 130-098-373, No. 130-100-825, Miltenyi Biotec, Bergisch Gladbach, Germany) are used according to the manufacturer recommendations. In brief, excised ventricles from mouse hearts at the age of one, respectively two, days postpartum are enzymatically digested. The cell suspension contains ventricular cardiac myocytes and other cardiac cell types (e.g., fibroblasts, endothelial cells, smooth muscle cells). The non-cardiomyocytes are depleted using a mixture of monoclonal antibodies coupled to MACSmicrobeads and subsequent retention in a column within a magnetic field. The column eluate contains the cardiac myocytes, which are seeded on patterned polymer surfaces without further preparation steps. The cardiac myocytes resulting from 1.5 murine neonatal hearts are used to cover one micro-patterned polymer field.

For hIPSC-derived cardiac myocytes terminally-differentiated Cor4U CLfresh cells were purchased from Axiogenesis, Cologne, Germany. On the day of shipment, 5 × 10${}^{5}$ cells are removed from a T25 bottle by conventional trypsin digestion and used to cover six 144 mm${}^{2}$ polymer-patterned and fibronectin-coated FluoroDishes using the manufacturer’s culture medium supplemented with 25 mM HEPES (Carl Roth GmbH, Karlsruhe, Germany). No further passaging is required, because the cells divide rarely.

Both cell types are kept at 37 ${}^{\circ }$C in water vapor-saturated atmosphere with 5% CO${}_{2}$. The neonatal cardiac myocytes are kept in culture for up to 48 h, and the hIPSC-derived cardiac myocytes are used from 24 h up to six weeks after passaging with medium change every 48 h.

### 2.3. Immunostaining, F-Actin and Nuclear Stain

If not otherwise stated, all chemicals are from Life Technologies GmbH, Darmstadt, Germany, and phosphate-buffered saline (PBS) is used as the solvent. For further preparation, the cells are briefly rinsed with PBS supplemented with 1 *μ*M calcium and 2 *μ*M magnesium at 37 ${}^{\circ }$C and fixed in 4% formaldehyde for one hour. All antibodies are diluted in PBS without calcium and magnesium supplemented with 1% (*w*/*v*) bovine serum albumin Fraction V, (ethanol precipitated, fatty acid free) (No. 8806, Sigma Aldrich, Munich, Germany) and 0.1% sodium azide (Merck GmbH, Darmstadt, Germany). Phalloidin labeled with Alexa-647 (1 *μ*g/mL) and DAPI (50 ng/mL) is dissolved in demineralized water with 0.1% sodium azide. All washing steps are performed in PBS. Specimens are kept for storage in PBS with 0.1% sodium azide. The following antibodies are applied in a 1:100 fold dilution: mouse monoclonal anti-*α*-actinin primary antibody (No. A7811, Sigma Aldrich, Munich, Germany), rabbit polyclonal anti-connexin-43/GJA1 primary antibody (No. ab150061, Cambridge, U.K.), goat polyclonal anti-mouse secondary antibody conjugated to Alexa-555 (No. 150118, *ibid*.) and donkey polyclonal anti-rabbit secondary antibody conjugated to Alexa-488 (No. 150061, *ibid*.). The consecutive order of application is: primary antibodies anti-*α*-actinin (AB-I) followed by secondary antibodies anti-AB-I. Intermediate fixation was performed in 2% formaldehyde solution at room temperature for 1 h. Primary antibodies anti-connexin-43/GJA1 (AB-II) are followed by secondary antibodies anti-AB-II. Phalloidin application was followed by DAPI application. Every application step took 12 h at 4 ${}^{\circ }$C followed by 5 times washing for 5 min at ambient temperature.

### 2.4. Confocal Laser Scanning Microscopy

The Leica TCS SP5 DMI6000 (Leica Microsystems, Germany)inverted microscope is used with an HCX PL APO lambda blue 63x 1.4 Oil UV-type objective lens. Four fluorescence acquisition windows (from I to IV) and one bright field transmission window (V) are defined as follows: I, excitation 405 nm, acquisition 414–483 nm for nuclear DAPI fluorescence; II, excitation 488 nm, acquisition 501–551 nm for connexin-43/JGA1 Alexa-488 fluorescence,; III, excitation 561 nm, acquisition 570–623 nm for *α*-actinin Alexa-555 fluorescence; IV, excitation 633 nm, acquisition 642–732 nm for f-actin phalloidin Alexa 647 fluorescence; V, excitation with accumulated excitation, specimen absorbance acquisition. The frame size is 246 by 246 *μ*m with 2048 by 2048 pixel discrimination, and the pinhole diameter is fixed at one airy unit (equals 95.5-*μ*m aperture sizes). Simultaneous channel acquisition in one-directional scan mode with eight-fold line averaging resulted in a total pixel dwell time of 80 *μ*s. Signal calibration is done by photomultiplier tube (PMT) detector excitation using the manufacturer’s calibration look-up table (Leica green -> glow-red -> blue LUT). Each channel recording is exported as an 8-bit grey scale uncompressed image in tiff format. Color overlays are done in ImageJ. If necessary, nuclear-located connexin-43 signals have been removed via channel-based pixel-wise signal subtraction using the DAPI recording as the subtraction template in the connexin-43 recording. The subtraction is done via the standard paste control function with option subtraction modifying the paste action in ImageJ (National Institute of Health, Bethesda, MD, USA).

### 2.5. Scanning Electron Microscopy Preparation and Recording

For SEM recording, formaldehyde-fixed specimens are post-fixed with 4% glutaraldehyde in PBS for one hour at 20 ${}^{\circ }$C. Further staining steps are performed in demineralized water. The specimen is incubated with each of the following solutions at ambient temperature for one hour and is subsequently washed out by a 5-step washing cascade: 1% osmium tetroxide followed by 1% tannic acid and, finally, 1% osmium tetroxide. The specimens are desiccated in an ascending dilution series of ethanol p. a. in demineralized water (dilution steps: 10, 25, 50, 75, 90, 2 × 100%, each step for 10 min) and air dried at 50 ${}^{\circ }$C followed by a 15-nm gold sputter coating and SEM recording. Critical point drying (CPD) is not applied due to swelling of PDMS in the intermediate fluids acetone or amyl acetate used to facilitate the exchange of ethanol against liquid carbon dioxide. Without an intermediate fluid, it is complicated to substitute ethanol with carbon dioxide. Such a risk for possible shrinking crackles must be calculated against the possibility to destroy the surface patterns by PDMS swelling in an inappropriate solvent, like acetone or amyl acetate. SEM images are recorded at 20-kV acceleration voltages using a backscatter electron detector. The use of this detector results in an aspect perpendicular to the specimen plane and an isotropic distribution of charging halos around the specimen edges. Differently, a secondary image detector would produce asymmetric halos due to the geometric angle between the incident electron beam and the observation vector of the detector. The working distance is 15 mm with a 500-fold magnification and spot size of 5 nm. The recorded images are 2048 by 1887 pixels, 8-bit grey scale in uncompressed tiff format.

### 2.6. Real-Time Recording of Cardiac Myocyte Contractions in Line Pattern-Organized Ensembles

The coordinated contraction of multicellular cardiac myocyte ensembles was acquired at a video frequency under two different contrast modes. First, an unstained culture was imaged in bright field illumination with a tilted illumination source and a partially-covered intermediate image plane. The anisotropic illumination resulted in shadows sketching the line pattern and the cell borders. Second, a vital dye, the Calcium Green-2in the form of an acetoxymethyl ester, was loaded to the cytoplasm. Upon excitation with blue light ($\lambda_{ex.}$ 450–490 nm), green fluorescence can be recorded ($\lambda_{em.}$ 510–550 nm). The calcium-sensitive fluorescence of the dye is superposed by strong baseline fluorescence. This fluorescence is used to record the mechanical cell contraction with a high signal-to-noise ratio. The trace was collected by the intensity projection of a specimen location across an image stack collected at 25 pictures per second. The region of interest (ROI) was placed beside a local fluorescence dye accumulation. Every contraction pulled the accumulation into the ROI and, thus, increased the average ROI signal. Upon relaxation, the fluorescent cell area shifted out of the region of interest, and the recorded intensity signal decreased. The recordings could be used to show that hIPSC-derived cardiac myocytes contract under the influence of a surface pattern in a spatially-coordinated manner (Figure 4g). The contraction frequency approximates the frequency of cardiac myocytes derived from conventional heart preparations.

## 3. Results and Discussion

**Figure 1:**
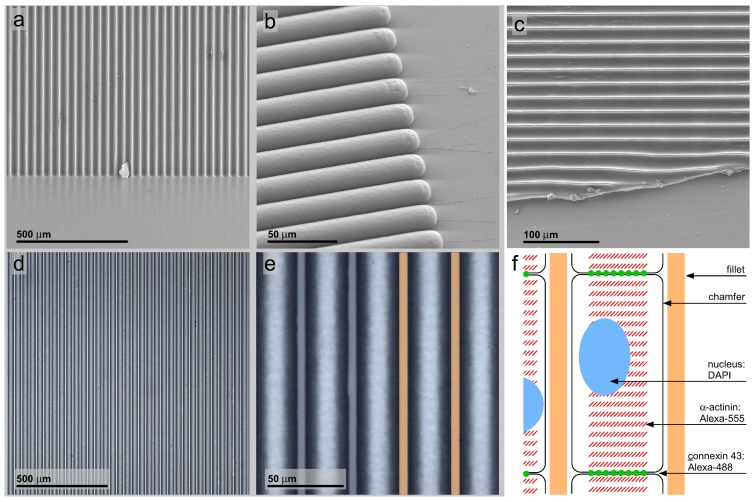
Matrix and stamp intermediates in patterned polymer production. (**a**) SEM recording of microlithography etched silicon wafer matrix for PDMS negative stamp molding; (**b**) stamp negative molded from the matrix in PDMS polymer; (**c**) a patterned PDMS polymer sheet on glass replicated by pressing the negative stamp into a thin uncured polymer layer and peeling it off after heat curing; the sheet is cut manually to demonstrate the polymer thickness; (**d**,**e**) bright-field light microscopy recordings of the polymer sheet; note the high regularity and precision over a wide surface area; the yellow bars indicate the 5 *μ*m-wide convex fillets alternating with 30-*μ*m concave chamfers; (**f**) sketch of the cell organization inside the surface pattern and the cell compartments stained for confocal laser scanning microscopy.

**Figure 2:**
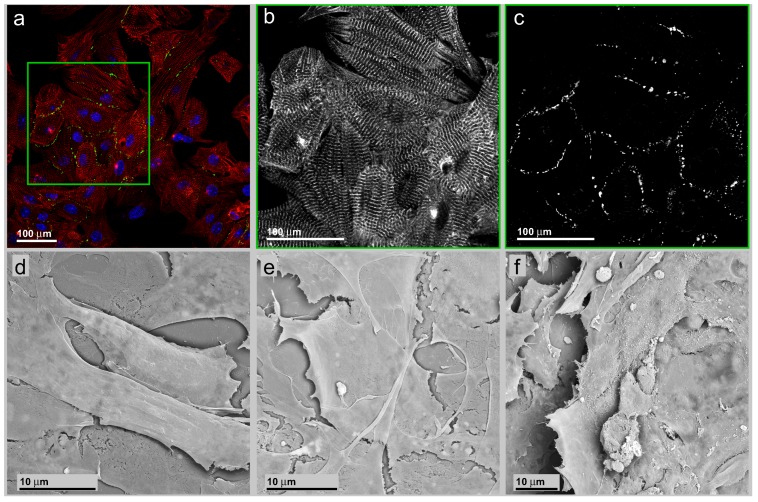
(**a**) Survey of a group of neonatal murine cardiac myocytes on a smooth glass substrate. The cells are stained for nuclei (blue), gap junctions via connexin-43 immunostaining (green) and the contractile apparatus via *α*-actinin immuno-staining (red); (**b**) (insert in (a))Exclusive presentation of the *α*-actinin signal component; (**c**) Exclusive presentation of the connexin-43 signal component. Besides the accumulation along pearl chain-like structures located along the intercellular borders, a faint non-specific signal is also visible inside the nuclei due to a cross-over in the acquisition channel. (**d**)–(**f**) are SEM recordings of neonatal cardiac myocytes cultivated on plain glass surface. The cells are flat and polygonal with an irregular polygonal shape.

**Figure 3:**
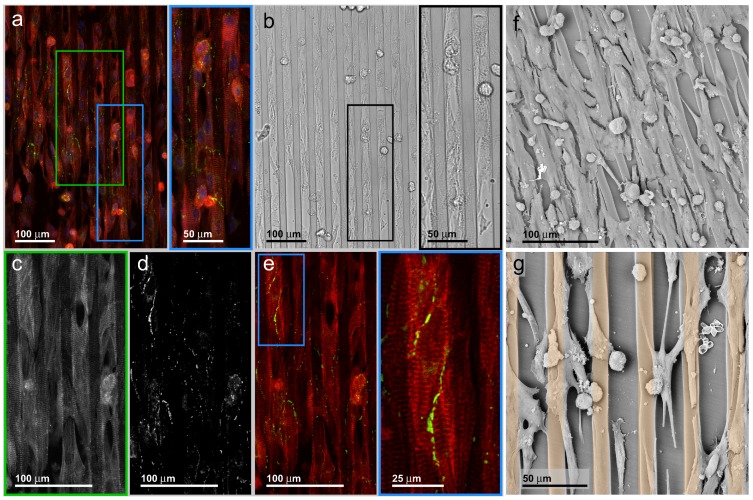
Fluorescence (**a**) and bright-field (**b**) light microscopy recordings of a typical murine cardiomyocyte specimen situation. A multicellular ensemble in near-confluent cell density is orientated along the polymer line pattern series; (**a**,**c**) (red and green inserts) Dual color overlay of the intracellular *α*-actinin (red) distribution and the intercellular connexin-43 distribution (green). The red signal closely follows the cell shapes, indicating that all cells are densely filled with *α*-actinin. Image (c) represents a higher magnification of the green rectangle with the *α*-actinin recording; (**d**,**e**) (blue insert) Distribution of the gap junction protein connexin-43. The pearl chain-like accumulations are located at the cell interfaces inside the cell contacts. A more homogeneous distribution of small signals all over the cytosol indicates non-recruited protein; (**f**,**g**) SEM recording of murine cardiac myocytes cultivated on line-patterned substrate. Vital cells are orientated along the fillets, while a randomly-distributed residual of rounded cells indicates the loss of cells during preparation from heart tissue; (**f**) Near confluent inoculation does not impede the organizing influence of line patterns, but it reduces the ratio of long cell protrusions. Such protrusions are more apparent at low cell densities (g). The golden overlay in (g) hints at the chamfer component of the surface pattern. An abstract sketch of the alternating chamfer to line pattern is given in Figure 1.

**Figure 4:**
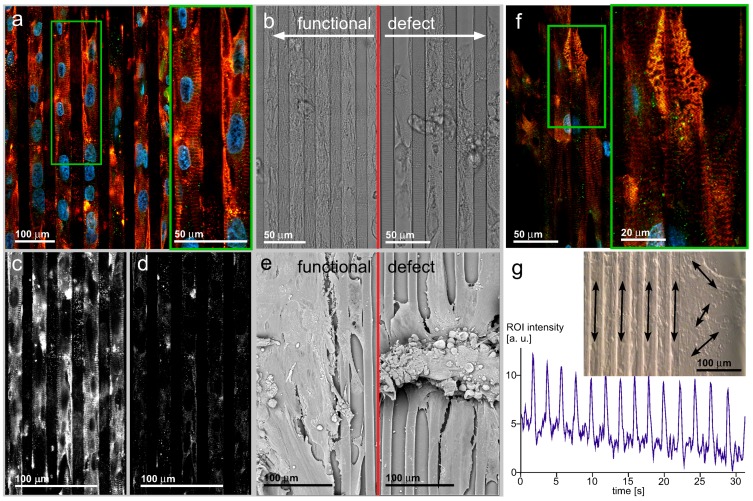
(**a**,**b**) (left) The long-term culture of hIPSC-derived cardiac myocytes yields cell assemblies similar to the culture of mouse primary cardiac myocytes, giving rise to the assumption that such stem cell populations can be used to substitute for primary animal-derived specimens; (**c**,**d**) Different from the well-ordered *α*-actinin apparatus, the connexin-43 distribution does not recapitulate the cell interface accumulation as seen in neonatal cardiac myocyte culture; (**e**) Functional (left) and defect (right) cell ensembles imaged by SEM. The obvious finding that the well-spread cell ensemble is a functional one while the defect one releases surface contacts and reaches a bulk appearance does not absolutely correlate with a loss of contractility. A loss of surface-mediated organization can be regarded inside the bulk assembly. Consequently, spatially-random contracting bulks can be observed; (**f**,**b**) (right) Aging effects of the contractile apparatus after long-term cultivation. The regular *α*-actinin organization with a ladder appearance with pronounced orientation along cells’ longitudes is substituted by a random orientation; (**g**) Ensembles of cardiac myocytes beat synchronously, indicating electrical excitation coupling. While the direction of contraction is random on a plain surface (right), the line pattern-induced cell ensemble organization results in temporally synchronized and spatially-organized contractions. This fulfills a fundamental prerequisite of cardiac tissue function.

In order to find appropriate geometric constraints, cell carriers with a line pattern were prepared on the basis of a microlithography etched silicon wafer (Figure 1a). This stamp was used to form a PDMS negative molded surface (Figure 1b) from which patterned PDMS line-shaped surfaces on glass cover slides were prepared (Figure 1c). The quality of the mold cast line patterns was controlled by light microscopic and scanning electron microscopic inspection (Figure 1d,e). In contrast to a non-patterned flat glass substrate, the cells should arrange along these lines, leading to electrical and mechanical coupled pearl chain-like cell ensembles, as schematically shown (Figure 1f). Mouse neonatal, as well as hIPSC-derived cardiac myocytes were grown on flat glass cover slides (only neonatal cardiac myocytes are shown; Figure 2) and line-patterned PDMS surfaces (neonatal cardiac myocytes, Figure 3; hIPSC-derived cardiac myocytes, Figure 4).

### 3.1. PDMS Micro-Patterning on Glass Substrates, Surface Post-Processing and Cell Seeding

The properties of the line patterns required for use in cell ensemble organization can be divided in three classes: efficient microlithography and polymer production, high optic quality and good cell compatibility. A pattern periodicity of 35 *μ*m with a minimum detail size of 5 *μ*m is well in scope of conventional microlithographic techniques. Consequently, the silicon micropattern is of sufficient quality (Figure 1a). By etching conditions, isotropic removal of silicon is done, resulting in a crescent shape of the chamfer with the maximum structure depth being half the maximum structure width. The etched silicon surface is smooth and facilitates the PDMS stripping after curing without structure damage for many times (Figure 1b). Up to 30 stamps could be molded from a silicon micropattern.

The negative PDMS stamp patterns molded from the silicon chips and also the PDMS replicas on glass carriers accurately preserve the three-dimensional pattern properties (Figure 1b,c). By light microscopy observation, the pattern shows only a few minor structure failures (Figure 1d). Even more important, the PDMS bulk is transparent and colorless, and the refraction index closely approximates the refraction index of the glass carrier used.

The PDMS patterns are plasma coated with a transparent colorless and non-fluorescent 15 nm-thick titanium dioxide (TiO${}_{2}$) layer. This surface coating makes the surface better wettable and decreases hydrophobicity. A high wettable is important for two purposes: the initial coating with water even in the line chamfers and the adhesion of cell adhesion facilitating proteins. After TiO${}_{2}$ coating, the patterns are immersed in a vitronectin solution. The resulting vitronectin layer improves further the adhesion of cardiac myocytes. Consequently, additional procedures during cell seeding are not required, and the seeded cells adhere in less than four hours.

### 3.2. Neonatal Mouse Cardiomyocytes on Unstructured Surfaces

The heart ventricle muscle of newborn mouse, as well as the neonatal human heart ventricle are highly organized on the tissue scale. Upon enzymatic digestion to release individual cardiac myocytes from the tissue bulk, the spatial organization is lost and cannot be reconstituted by cultivation on plain surfaces. This covers both the multicellular organization, as well as the intracellular setup of the contractile apparatus.

To explore the cardiac contractile apparatus, *α*-actinin staining can be used. The *α*-actinin composition and distribution correlates well with the regular contraction activity and also with the maturation state of cardiac myocytes [29]. During the derivation of cardiac myocytes from hIPSC cells and a subsequent maturation process over several weeks, the contractions synchronize. The contraction amplitude increases, while the contraction cycle length variation decreases [30,31]. This makes the *α*-actinin a good marker for the contractile maturation status of hIPSC-derived cardiac myocytes. In murine neonatal cardiac myocytes, *α*-actinin is reported to be almost fully developed and, thus, can be compared to that in hIPSC-derived cardiac myocytes [32].

Connexins (mainly connexin-40, connexin-43 and connexin-45) are proteins that form the intercellular gap junctions. The composition ratio of the mentioned connexins, but also the turn-over, the plasmalemmal density and accumulation, modulates their electrical signal transduction properties [33,34]. In cardiac tissue, this is of great importance for spreading of the electrical signal yielding to the typical electrical syncytium. Mismatches in connexin handling and a subsequent irregular gap junction status correlate with common cardiomyopathies, such as hypertrophy [35,36]. Conversely, a congestive or acquired connexin handling failure can be the source of cardiac arrhythmias and increase the probability of life-threatening ventricular tachycardia [37]. This makes the connexin-43 distribution an interesting parameter for a cell system that is in dynamic development [38,39]. This is of even higher importance if the cell population shows an inhomogeneous maturation state with differing action potential characteristics, which usually occurs due to a lack of optimal differentiation protocols [39,40].

In the case of cell culture-derived cell systems, the gap junctions, as well as the contractile *α*-actinin contraction system are newly-generated, located at or influenced by the intercellular contact lines, but their pattern does not reflect an ordered signal spread and the concerted forces’ interaction over several cells.

In Figure 2a, a survey on a group of neonatal cardiac myocytes on smooth glass substrate is presented. The cells are stained for nuclei (blue), gap junctions via connexin-43 immuno-staining (green) and the contractile apparatus via *α*-actinin immune-staining (red). The local cell accumulation, as well as the individual cell orientation is random. The well-ordered intracellular organization of *α*-actinin in sarcomere structures reflects the high degree of organization of the contractile apparatus (Figure 2b).

The organization further facilitates the identification of local directions of cell contraction. Neither inside an individual cell nor between neighboring cells in the ensemble does a commonly-organized contraction appear. This does not exclude the possibility of local synchronous contraction. The cell ensemble does not develop coordinated beats over longer distances with a common direction, also different from the tissue state. The distribution of connexin-43 signals is primarily restricted to the intercellular contact lines without accumulations inside the cytoplasm and recapitulates the native organization of gap junctions in cardiac tissue and cell aggregates after extraction from cardiac tissue [41]. Besides the accumulation along pearl chain-like structures located along the intercellular borders, a faint signal inside the nuclei appeared (Figure 2c). This was interpreted as a result from an acquisition channel cross-over due to the extended spectral emission of DAPI nuclear staining. The subtraction of the corresponding DAPI image from the connexin-43 recording removes the nuclear-located connexin-43 signal exhaustively while leaving the cytosolic connexin-43 signals untouched. An accumulation of connexin-43 protein in the nucleoplasm is not to be guessed, because of the endoplasmatic reticulum membrane-restricted character of the transmembrane domains.

In Figure 2d–f, SEM recordings from neonatal cardiac myocytes cultivated on smooth glass surfaces are shown. The cells are widely spread, flattened and of a polygonal shape (Figure 2d). This is in contrast to the native tissue situation with cardiac myocytes shaped as elongated cubical. It also contrasts with the shape of the same cells cultivated on patterned surfaces showing a more tissue-like cell shape (Figure 3c). Resembling one tissue characteristic, the cell ensemble consists of several cell layers, indicating that in a non-tissue environment spatial contact inhibition does not appear (Figure 2e). The loss of contact inhibition can be observed at cell ensembles of both low (Figure 2e) and high (Figure 2f) density.

### 3.3. Neonatal Mouse Cardiomyocytes on Line-Patterned Surfaces

In contractile heart tissue bulk, the location and shape of an individual cell is determined by the locations and shapes of the surrounding cells. As an immediate consequence, the organization of the contractile *α*-actinin-containing apparatus and the intercellular gap junctions recapitulate this cell ensemble organization. In order to imitate such tissue-specific spatial cues by a regular patterned environment, it is of high interest to examine the *α*-actinin and also the connexin-43 organization with respect to cell ensemble alignment and individual cell shape. Hereby, the long-term stability of the *α*-actinin will give a structure-based measure for the development of the contraction power-generating apparatus over time, while the connexin-43 provides information about the electrical signal spread.

Fluorescence (Figure 3a) and bright-field (Figure 3b) light microscopy recordings of a typical specimen situation can be obtained on line-patterned carriers. The fillet chamfer pairs appear as vertical ribbons in both recordings. The chamfers are filled with cardiac myocytes, while the fillets are cell-free over long distances. This cell distribution is an effect of the chamfer geometry, mainly caused by the maximum width near a cell diameter and the concave shape limited by a convex edge. The local transition from concave to convex surface topography imposes growth directions on migrating, as well as contracting cells. This results in a cell-directing property of the topographic patterns. The bright-field image further illustrates the individual cells being elongated with the cells’ length axes in parallel to the pattern direction. The spatial organization of the contractile apparatus, as well as the gap junction distribution is organized by the overall cell orientation. This indicates structural adaptation of the individual cell and the cell ensemble, an organization that is induced by the pattern topography. In comparison to Figure 2, the surface pattern suppresses the tendency of ensembles consisting of individual cells to orientate and shape in a random fashion. Oppositely, the pattern stimulates the geometric orientation of any individual cell and concentrates cell ensembles at certain positions, mainly inside the chamfer areas.

The line-pattern not only shapes the orientation of the cells, but also forces cells to shape into cylindrical geometries. Figure 3a displays a dual color recording of the intracellular *α*-actinin (red) distribution and the intercellular connexin-43 distribution (green). The red signal closely follows the cell shapes, indicating that all cells are densely filled with *α*-actinin.

Figure 3c represents a higher magnification of the green rectangle with exclusively the *α*-actinin recording. The protein shows a characteristic cross-striation patterning with the main direction of contraction organized in parallel to the external chamfers’ topography. Cells with disorganized *α*-actinin were not abundant. Possible explanations for this finding could be the high ratio of cardiac myocytes in the cell preparation or a high vitality over the culture period. In contrast to the bright-field image, cells spanning the fillets can be seen. This difference might be an effect of the higher signal-to-noise ratio of fluorescence recordings as compared to bright-field recordings. Furthermore, the cardiac myocytes spanning the fillets are orientated along the patterns’ length axes and like the cells located inside the chamfers organizing the contractile apparatus along the cell length axis. This indicates that even a closed monolayer favors a geometrical organization in accordance with the external geometry.

This is also reflected by the distribution of connexin-43 (Figure 3d), which concentrates along the cell borders and appears in small foci, as required for gap junctions. The distribution of the connexin-43 foci is anisotropic: more foci chains appear inside the chamfers than across the fillets. This finding leads to the assumption that the electrical coupling of cardiac myocytes in the ensembles and, consequently, the spread of an electrical signal follow the external surface topography. For the spatial distribution of contraction forces, analogous assumptions can be made (see below and in a movie given in the Supporting Material).

The distribution of the gap junction protein connexin-43 must be regarded to fall into two subpopulations, *i.e.*, protein recruited to gap junctions and non-recruited protein inside the cytoplasm. The second subpopulation distributes in low density over the entire cell interiors, while the first subpopulation accumulates inside dense foci. On the basis of the current recordings, the direct identification of cell borders is not possible, due to the lack of a cell membrane-specific marker (Figure 3e). In future experiments, the use of fluorescently-labeled wheat germ agglutinin as a cell surface marker could address this need. As the images, due to their confocal recording, represent thin lateral optical slices from a cell bulk with a non-negligible axial extension, two effects have to be considered for the gap junction recruited connexin-43 protein population. First, the number of protein foci may be higher than shown in the image, because the foci outside the recording plane are suppressed. Z-stack recordings would be helpful in elucidating this distribution pattern, but the evaluation of the spatial dataset requires a certain instrumentation of analysis methods, making this an autonomous approach not to be covered here. Secondly, the erratic distribution of foci along undulating strings might be a result of the cell ensemble organization. Different from a plain 2D substrate, where cell contacts are restricted to cell contacts on a flat surface, the chamfer surface folding along a semi-circular shape increases the cell surface area prone to gap junction formation. This way, the patterned surface approximates the spatial situation in 3D tissue more closely than a plain substrate.

Scanning electron microscopy (SEM) recording of another specimen with comparable cell density was performed (Figure 3f,g). Different from the bright-field image or the confocal image, the SEM recording facilitates the identification of cells spanning over the fillets. Two opposing findings can be formulated. The first finding is that a substantial ratio of isolated cells, as well as cells in ensembles is capable of spanning the fillets and to cover more than one chamfer. In theory, this should drive the cells to lose their parallel orientation as induced by the substrate pattern and to organize in random orientation. However, a close examination of the elongated cells shows them preferentially orientated along the pattern geometry. This means that even cell ensembles, usually displaying internal mechanical feedback during growth and migration, are under the topological control of the substrate pattern.

### 3.4. hIPSC-Derived Cardiac Myocytes on Line-Patterned Surfaces

The hIPSC-derived cardiac myocytes have been cultivated for six weeks on the patterned substrate (Figure 4a–f). The organizing property of the surface pattern was conserved over the cultivation period, prohibiting the disorganization of the contractile apparatus and the cell shape. The increased cultivation time induced local defects in the *α*-actinin organization. Accumulation of connexin-43 in gap junctions cannot be shown, but all cells of an ensemble contract simultaneously. This is possible by electromagnetic potential jumps generated by local autonomous exciting cells and inducing cell depolarization in closely-packed cell assemblies [26].

**Figure A1:**
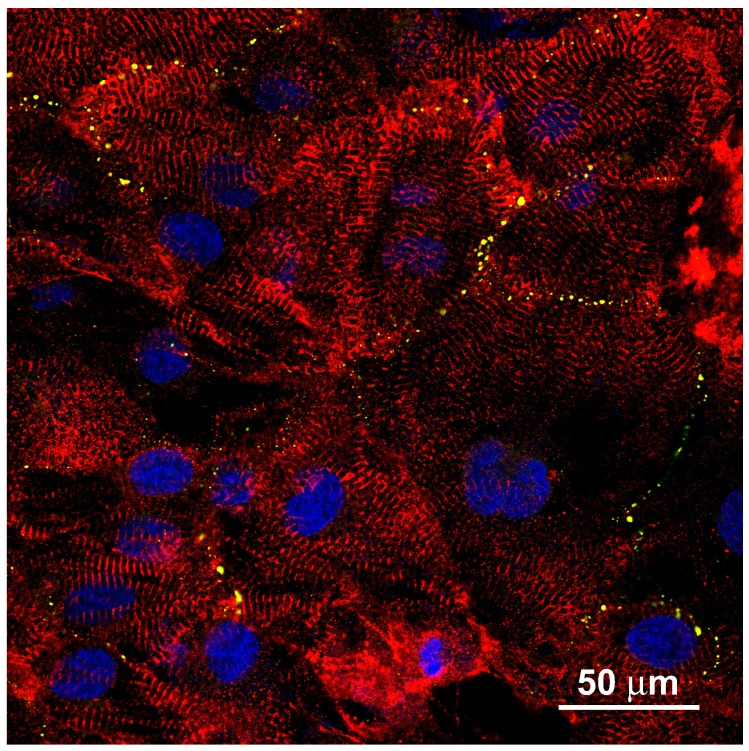
hIPSC-derived cardiac myocytes grown on a plain glass substrate are very similar to mouse cardiac myocytes shown in Figure 2. Cell shape and intracellular *α*-actinin organization (red) are only locally regular, but disorganized inside individual cells and across the cell group. Gap junctions (green) accumulate in dots at the cell contacts. This fulfills a fundamental prerequisite of cardiac tissue function.

The overall appearance of a cardiac myocyte hIPSC-derived monolayer closely resembles the situation found in the mouse cardiac myocyte ensembles (Figure 3a,b for line patterning; Appendix Figure A1 for unpatterned surface). The cells are organized in high density along the chamfers. The bright-field image shows them covering also the fillets. Despite the confluent cell density, the individual cells arrange in parallel to the pattern geometry. The insert in Figure 4a shows the high degree of organization of the contractile apparatus. Counting of cell nuclei allows a rough estimation of the cell number and, thus, indirectly, an estimation of the cell size without exact localization of cell borders. For this estimation, it must be considered that bi-nucleated cells can appear and influence the resulting cell number. The cells are 80–100 *μ*m in the longitudinal direction and of 35–40 *μ*m transversely. Different from the well-organized contractile apparatus, the connexin-43 distribution remains homogeneous without focal accumulations, as required for gap junctions (Figure 4c,d). The appearance in small spots rather than in the form of an unstructured background makes recruitment into just small protein complexes likely.

During the extended cultivation time, the cells accumulate in 3D bodies with loose surface contact. The bright-field image shows a typical situation of a cell ensemble. The periphery is filled with a monolayer of spread cells, while in the center, bleb-like structures appear. In such bodies, confocal microscopy identifies disaggregation states of the contractile apparatus. The disorganized cell ensemble together with disaggregation of the ladder patterns can result from the loss of mechanical force, as present in cardiac muscle tissue. The *α*-actinin ladder develops branches and loses the close orientation to the cell length axis (Figure 4f).

Figure 4 shows SEM recordings of functional ensembles of hIPSC-derived cardiac myocytes and defect ensembles (Figure 4e). While the bright-field images indicate how the cells fill the pattern, the SEM recordings reveal that the cells are also spread above the chamfer level and in close contact across the fillet borders. Regardless of the isotropic distribution of cell to cell contacts, the individual cells inside the ensemble conserve the main direction of the underlying pattern geometry. It may be deduced that the electrical, as well as the mechanical coupling follows this principle; and the ensemble contraction is synchronized not only along an individual chamfer longitudinal space, but the cells synchronize over the fillet borders. This assumption is confirmed by video sequence acquisition of beating ensembles on patterns with a prominent main direction of contraction in a temporally-synchronized manner. The defect ensemble on the right shows a central cell bulk without organization. This is also apparent in bright-field. The bulk sits on top of a residual cell ensemble with individual cells orientated along the chamfer longitudinal direction. Such cells are contractile, as shown in the video recordings.

Figure 4g shows the appearance of a living ensemble of hIPSC-derived cardiac myocytes organized by an underlying pattern. The video recording (refer to the Supplements) shows the overall direction and beating synchrony, while the trace in Figure 4g shows the beating frequency over time. The trace reflects the contraction of a location inside a cell ensemble over a 30-s time window. The beating frequency is slightly below 0.5 Hz and remains constant over the observation time. The bright-field image in the insert represents a video sequence of a corresponding cell ensemble cultivated for six weeks on a patterned surface. The pattern edge facilitates the comparison of beating cell ensembles with and without an underlying pattern. The organizing influence of the cell contraction reflects the findings of the *α*-actinin immuno-fluorescence. According to the high organization level of the contractile apparatus along the pattern, the cells closely contract along the chamfer to fillet periodicity. In contrast, on a plain substrate (right), local contraction centers develop individual, non-orchestrated beats with several directions.

## 4. General Discussion

In the present work, we stimulated a high number of ensembles of individual cells to organize themselves in a tissue-like fashion. Both cell types under examination, mouse neonatal cardiac myocytes and hIPSC-derived cardiac myocytes, were organized similarly to the situation in ventricular muscle [32,42]. Their organization was controlled by a regular pattern of alternating concave chamfers and convex fillets with a periodicity in the regime of a single cell diameter. With a high ratio, not only the outer cell shape adapted to the pattern motive, but also the inner cell organization and the intercellular contacts have been organized by the patterned surface. The organizing effect can be seen in the cell orientation, the intracellular contraction apparatus and the intercellular gap junction accumulation.

### 4.1. Statistical Aspects of the Parameters Observed

Reliable parameters representative for the transition from cardiac myocyte precursors into mature cardiac myocytes utilize changes in the expression pattern of two TNNI family genes, the TNNI1 gene expressed in the developing heart and the TNNI3 gene, which is active in the mature heart. During development, a quantitative switching between the two gene isoforms can be detected [43]. Such a parameter can be used to test a high number of developing cardiac myocytes, but an examination of a small number of cells is not possible. Furthermore, geometrical properties of the approach presented here cannot be addressed. Alternatively, the statistical analysis of gap junction dimensions in myocardial tissue by transmission electron microscopy elucidates some basic problems related to the determination of numerical values. A number of corrections have been introduced to compensate for acquisition-related failure sources, like specimen tilt or local deformation [44]. The corrections resulted in the decreased validity of the values. Such considerations motivated us to state our findings on selected representative specimen situations. Nevertheless, our findings represent a reasonable number of cells organized on a large line-patterned area of 12 × 12 mm. This area includes 342 chamfer to fillet pairs with a periodicity of 35 *μ*m and a length of 12 mm. A realistic cell long axis can be averaged to 60 *μ*m with a chamfer fill factor of one, *i.e.*, cells inside a chamfer organize in a pearl chain fashion. By appropriate high seeding density, a confluent monolayer is reached, and the differentiation character of the cells makes it sure that the seeding density is not biased by cell division. The total chamfer length of a PDMS mold is $342\times 1.2\times 10^{4}$*μ* m or 4.104 m. This is sufficient chamfer space to host 68,400 60 *μ*m-long cells. The microscopic field of view covered a 387 *μ*m square. This results in a total chamfer length of 2723 *μ*m hosting 44 cells at confluency. For each line pattern situation described, we analyzed a minimum of 20 non-covering and non-touching specimen locations. This resulted in a number of 880 cells analyzed in total per experiment situation.

### 4.2. Line Patterns as Modular Elements in Cardiac Tissue Engineering

Our approach addresses an essential demand in tissue engineering, namely the organized arrangement of cells of a designated type into the spatial order prescribed by the tissue of interest. In this sense, our approach opens up a way towards a promising use of stem cell-derived cell types to reconstitute functional tissues and organs. In the present case, the pattern closely stimulates the line-wise arrangement of cardiac myocytes, resulting in a working myocardium tissue slice imitation. In contrast to tissue slices from a donor heart, our approach has some advantages: we have no cell debris from cells destroyed during the slicing procedure. By a computer-aided design, we can precisely define the orientation and density of the line pattern and, this way, set up artificial slices with a well-defined cell orientation. By analysis of conventional histological slices, the geometrical constraints can be adapted to any required tissue type. Currently, the resulting tissue slices are only two-dimensional, but a three-dimensional tissue bulk is accessible via cell sheet engineering [17]. The surface geometry serves as a constraint for cell shaping and arrangement. Regular alternating chamfers and fillets result in a geometric surface pattern with a periodicity close to the native cell diameter. In the case that the pattern interacts with the migration activity of the individual cells, the ensemble is orientated in line-wise fashion with the cells cueing up inside the chamfers and being in frame line by line. Our geometry pattern is highly anisotropic and highly regular. This imitates a prominent geometric motive of cardiac ventricle tissue. The preparation procedure ensures that the cell interaction is exclusively mediated via pattern geometry and not by cell influencing proteins. The approach mentioned above defines a type of elementary cell consisting of a concave to convex transition in the cell size regime. This basic element can be multiplexed to set up regular, as well as irregular surface areas. Such areas can recapitulate the appearance of tissue slices [16]. This approach will stimulate activities in the generation of artificial tissues and organs. Advanced research fields, like stem cell-based tissue engineering and tissue sheet engineering, as well as pharmaceutical compound safety testing and radiation research, are touched upon [17,45].

### 4.3. Pattern Geometry Mechanically Stimulates Cell Orientation via Interacting Adhesion and Contraction Behavior

The organization of cell populations by cell restricting geometrical motives is reported repeatedly [14,46,47,48]. If line-patterned surfaces are in use, the active line periodicities range from the nanometer to the micrometer regime, and the postulated pattern action covers the arrangement of adhesion complexes or the overall cell adhesion dynamics [15,49]. In our work, we define a line-patterned surface as a repetitive alternation of concave (chamfer) and convex (fillet) surface increments; the size of either is a cell diameter (concave) or a typical protrusion zone (convex). The underlying functional principle is the interaction between cell-to-surface complexes, like focal adhesions and cell tension, generating cytoskeletal elements, like f-actin fibers. In their comprehensive theoretical approach, Maraldi and coworkers formulated optimal ratios between adhesion and contraction cell activity [50]. Based on this formulation, our periodic pattern serves as a surface that positions an individual cell to make a decision based on different ratios of surface adhesion protein complexes and cell tension-generating cytoskeleton elements. On our surface patterns, the optimal ratio can be approximated by an individual cell when the cell body contacts the substrate in concave areas. Consequently, cells reside in the chamfers and do not overlap the fillets. Orthogonal arrangements are also not favored, because parts of the cell body or the entire cell must accept an unfavorable convex cell tension.

The inner cell organization and the establishment of cell interfaces were examined by imaging the *α*-actinin organization and the connexin-43 accumulation in gap junctions, respectively. For *α*-actinin, in all three examined situations, *i.e.*, neonatal mouse cardiac myocytes on plain *vs*. on patterned substrates and hIPSC-derived cardiac myocytes on patterned substrates, the conventionally-expected organization requirements are fulfilled [30,51]. A ladder-type organization indicates integration into the contractile apparatus. The random re-arrangement of *α*-actinin ladders during cardiac myocyte cultivation on plain substrates or the manifestation of outer shape geometries in the cytosolic *α*-actinin organization is a common phenomenon [52]. This loss of intracellular organization upon the release of an individual cell from the tissue bulk indicates a main problem in tissue engineering. With our approach, it is possible to stabilize the cytosolic organization of the contractile apparatus and to conserve spatially-orientated contraction as a main function of cardiac muscle cells. This conservation works over several weeks, as demonstrated by hIPSC-derived cardiac myocytes. The shown organization defects appearing locally as subcellular losses of z-disc regularity can be designated as maturation phenomena after a long cultivation period with suboptimal mechanical stress application [53]. They must be discriminated from the rapidly-proceeding disorganizations known from cardiac muscle cell populations on plain substrates and inside homogeneous scaffold bulks [7].

### 4.4. hIPSC-Derived Cardiac Myocytes in Long-Term Culture as Targets for Either Apoptosis or Incomplete Maturation

The non-canonical appearance of the contractile apparatus in an hIPSC-derived cardiac myocyte sub-population raises the question of the nature and reason for this constitution. In brief, two possible scenarios can be assumed. The phenomenon can result from a complex interaction of cell maturation, the accumulation of mechanical defects during continuous contractile activity or entrainment. While cell maturation can be reduced to intracellular physiological features, the accumulation of defects is a result of the interaction between the mechanical intercellular environment and the cell to surface connection pattern. Entrainment phenomena in contracting cardiac myocytes are known from embryonal heart development, as well as from spatial *α*-actinin organization plasticity during cultivation of cardiac myocytes in random cell culture. They manifest as local irregularities in contractile apparatus organization and can be reduced to mechanical reactions on a non-homogeneous force profile generated by the surrounding cells. The appearance of such disorganization sketches the organizing influence by the restricted surface patterns.

The nature of hIPSC-derived cardiac myocytes as a product of an artificial maturation process not fully controlled by a developing organism, but stimulated by a set of embryo-derived or newly-synthesized artificial compounds [54] raises the question of whether the development of an individual cell from an undifferentiated precursor into a highly differentiated heart muscle cell is recapitulated exhaustively. This consideration must also include whether any cell of a long-term cultivated ensemble has reached the same differentiation state or if a certain ratio of cells entered different cell fates [55]. The maturation state of cardiac myocytes has two main aspects. The first aspect covers the subcellular organization state, as analyzed in the present paper. The second aspect focuses on the cell activity as the development of excitability and contractility. The contractile aspect of maturation is examined in depth using *α*-actinin organization as the parameter. The cellular excitability is examined by the observation of concerted contractions across a multicellular ensemble. The methodology applied does not regard the dynamics of the membrane potential-generating protein set and its activity, because electrophysiological measurements are not performed [31,56].

### 4.5. Connexin-43-Containing Gap Junctions Require More Information for Correct Intercellular Localization than Patterned Surfaces Provide

The connexin-43 organization differs between the two cell types. The mouse neonatal cardiac myocytes develop connexin-43 accumulations along the cell borders, both on plain and patterned substrates. Isolated mouse cardiac myocytes do not develop comparable accumulations, indicating that the connexin-43 signal marks the gap junctions between adjacent cells. Gap junctions are an important component for the transduction of electrical excitation across cardiac myocyte ensembles [57,58].

The accumulation of gap junctions in the intercellular regions together with the cytosolic depletion indicates a native cytosolic connexin-43 protein trafficking. This finding is in clear contrast to the connexin-43 distribution in hIPSC-derived cardiac myocytes cultivated on lines, where the distribution of connexin-43 does locate only to a minor ratio at cell borders in the ensemble. This indicates the stochastic behavior of the gap junction constitution in cell ensembles in constraint-free cell culture environments and differs from the situation in native tissue with a presence of connexin-43 at all outmost intercellular contacts. We compare the connexin-43 organization in two cell systems, which differ substantially in the regulation of gap junction setup. The setup of gap junctions in the developing, as well as remodeling heart is controlled by ErbB receptor-dependent signal cascades. In particular, ErbB 2/4 dimers regulate the gap junction accumulation and capping upon binding their agonist neuregulin-1 [59]. While murine neonatal cardiac myocytes have been supported with neuregulin-1 during embryonic development, the hIPSC-derived cardiac myocytes are cultivated in a neuregulin-1-free environment. Blocking of neuregulin-1 action by therapeutic antibody trastuzumab is shown to disorganize gap junction organization and capping *in situ* [60]. Consequently, it is of great interest to examine the gap junction organization inside a tissue-like ensemble of cardiac myocytes lacking neuregulin-1.

In spite of this structural defect, video recordings of native hIPSC-derived cardiomyocyte living cells clearly show a coordinated contraction. Furthermore, Oyamada and co-workers report the establishment of gap junctions in hIPSC-derived cardiac myocytes [61].

### 4.6. Cell Organization by Line Patterns Influences Electrical Signal Spread via a Mechanism Different from Gap Junction-Based Depolarization Spread

The conduction of electrical signals across cardiac tissue is a highly organized process with the regular arrangement of individual cells and the gap junction accumulation at the cell termini (the cell caps) being the prerequisite for a controlled spatial signal spreading [62,63,64]. However, cardiac myocytes are also excitable by external electrical fields. Consequently, inside a dense cell ensemble, the local electrical potential jumps during spontaneous cell action potentials are sufficient to spread across the cells, resulting in synchronous beating. The handover of an electrical stimulus by fluctuation along a local electromagnetic field does not need a punch-through potential across gap junctions. It is independent of the blocking or activation state. During cardiac defibrillation, the application of gap junction blockers decreases the defibrillation threshold potential, indicating the reduction of potential flow across gap junctions being accompanied by an increase of the dielectric properties of the cardiac cells [65,66]. This addresses the question whether non-gap junction-supported electrical signal spread is a factor in addition to canonical spread across gap junctions, as recently speculated [26]. Electrical signal spread through dense ensembles of excitable cardiac myocytes requires potential jumps of the extracellular field, which depolarize cells comparable to the depolarization of a dielectric body inside a fluctuating field. This mechanism does not need intercellular signal spread via gap junctions. Lin and Keener describe such a type of signal spread across uncoupled, but densely-packed cardiac myocyte ensembles and ventricular tissue [26]. During the so-called ephaptic signal spread, electrical field jumps generated by a depolarizing myocyte induce depolarization in neighboring cells. This way, the excitation signal is handed over across tight intercellular spaces and not only via gap junctions.

### 4.7. Tissue Engineering and Pharmacology Drug Screening in an Organized Multicellular Environment

Tissue engineering greatly profits from recent advances in stem cell generation and differentiation of particular cell types. The high organization degree of differentiated cells in tissues and organs cannot be achieved under commonly-used culture conditions. Tissue of low organization complexity, like insulin-secreting islets [9,11], or random cell deposition followed by external gradient-based tissue organization, like unstructured myocardium [67], works satisfactorily. In clear contrast, the organization of regularly-composed tissues, like muscle or heart muscle, fails in the well-ordered organization of the differentiated cells [68]. Our approach will support this important aspect of artificial tissue and organ generation.

Drug screening also benefits from the availability of cell populations with both tissue-like organization and function. The action of a drug on an individual cell cannot recapitulate the action on a multicellular ensemble. Further, a random cell culture does not represent the spatial aspects of a native tissue. As a consequence, only a cell culture recapitulating not only physiological, but also the spatial aspects of the tissue present is capable of acting as a reliable template for compound testing. The long QTsyndrome is a case for the requirements described above. The long QT syndrome manifests as a tendency to develop ventricular arrhythmias, like torsade de pointes (TDP) [69,70], by a combination of sodium channel-induced early afterdepolarizations (EADs) and ventricular dispersion of repolarization (VDR) [71]. Finally, ventricular excitation pathway geometry results in the onset of self-sustaining cardiac arrhythmia [72]. To address this demand, the European Union initiatives S7/A and S7/B recommend the testing of any new compound with respect to arrhythmogenic capacity [25].

Currently, it is possible to derive cardiac myocytes with an LQTtendency from hIPSCs and to perform electrophysiology-based compound testing on an isolated single cell basis [73,74,75]. The onset of LQT-based arrhythmias has fundamental spatial aspects besides the electrophysiological behavior restricted to single cells [76,77]. Consequently, a reliable test either requires the use of laboratory animals or the use of artificial tissue sheets made by organized hIPSC-derived cardiac myocytes, which are prone to develop VDRs in a TDP-stimulating geometry of excitatory pathways. Our system holds great promises in addressing this key requirement.

hIPSC-derived cardiac myocytes are already in use as substitutes for cardiac myocytes from animal donors in personalized medicine [78]. Electrophysiological examinations using classical cell clamping electrodes for the action potential, as well as multi-electrode arrays (MEAs) for the spatially-resolved recording of field potentials show hIPSC-derived cardiac myocytes being promising targets for such purposes [79,80]. Especially the use of MEAs to record the field potential of tissue-like organized cardiac myocytes can identify the functional anisotropy characteristic for native tissue. This will be helpful in closing the gap between results of isolated cells and of cells in coupled ensembles.

## 5. Conclusions

In conclusion, primary cultures of murine neonatal cardiac myocytes and hIPSC-derived cardiac myocytes provide comparable results with respect to electrical signal transduction and coordinated contraction, even if differences in electrical signal transduction are apparent. The lack of gap junction establishment in hIPSC-derived cardiac myocytes is a promising area for future activities. The organized cultivation of both cell types on patterned surfaces simulates the tissue situation and extends the information value concerning the coordinated behavior of interacting cell ensembles. This approximation to the vital tissue organization is a step towards the replacement of animals as donors for organs and tissues. Furthermore, basic research will benefit from such an approach, because the close definition of geometric constraints in combination with identified cell types and a clear definition of the chemical and physical environment make it possible to reduce unpredictable specimen variations, being a typical restriction when working with donor tissues and cells. A closer definition of experimental parameters is also a prerequisite to install animal-free test systems for pharmacological research.

## Supplementary Files

Supplementary File 1

## Appendix A hIPSC-Derived Cardiac Myocytes on a Plain Glass Surface

For comparison, the appearance of hIPSC-derived cardiac myocytes grown on a plain glass surface is presented (Figure A1). The situation closely reflects the case of mouse embryonic cardiac myocytes freshly prepared from newborn animal hearts, as described above.
